# Comparison of Human and Soil *Candida tropicalis* Isolates with Reduced Susceptibility to Fluconazole

**DOI:** 10.1371/journal.pone.0034609

**Published:** 2012-04-05

**Authors:** Yun-Liang Yang, Chih-Chao Lin, Te-Pin Chang, Tsai-Ling Lauderdale, Hui-Ting Chen, Ching-Fu Lee, Chih-Wen Hsieh, Pei-Chen Chen, Hsiu-Jung Lo

**Affiliations:** 1 Department of Biological Science and Technology, National Chiao Tung University, Hsinchu, Taiwan; 2 Institute of Molecular Medicine and Bioengineering, National Chiao Tung University, Hsinchu, Taiwan; 3 National Institute of Infectious Diseases and Vaccinology, National Health Research Institutes, Miaoli, Taiwan; 4 Department of Applied Science, National Hsinchu University of Education, Hsinchu, Taiwan; 5 School of Dentistry, China of Medical University, Taichung, Taiwan; University of Minnesota, United States of America

## Abstract

Infections caused by treatment-resistant non-albicans *Candida* species, such as *C. tropicalis*, has increased, which is an emerging challenge in the management of fungal infections. Genetically related diploid sequence type (DST) strains of *C. tropicali*s exhibiting reduced susceptibility to fluconazole circulated widely in Taiwan. To identify the potential source of these wildly distributed DST strains, we investigated the possibility of the presence in soil of such *C. tropicalis* strains by pulsed field gel electrophoresis (PFGE) and DST typing methods. A total of 56 *C. tropicalis* isolates were recovered from 26 out of 477 soil samples. Among the 18 isolates with reduced susceptibility to fluconazole, 9 belonged to DST149 and 3 belonged to DST140. Both DSTs have been recovered from our previous studies on clinical isolates from the Taiwan Surveillance of Antimicrobial Resistance of Yeasts (TSARY) program. Furthermore, these isolates were more resistant to agricultural azoles. We have found genetically related *C. tropicalis* exhibiting reduced susceptibility to fluconazole from the human hosts and environmental samples. Therefore, to prevent patients from acquiring *C. tropicalis* with reduced susceptibility to azoles, prudent use of azoles in both clinical and agricultural settings is advocated.

## Introduction

Due to the elevated number of risk populations, the prevalence of fungal infections has increased significantly in past two decades. *Candida* species are the most frequently isolated fungal pathogens causing morbidity and mortality in seriously immunocompromised hosts. Although *Candida albicans* is the most prevalent species in hospitalized individuals and in nosocomial infections, there has been a shift toward the more treatment-resistant non-albicans *Candida* species [1], [2], [3], [4], [5]. This has become an emerging issue in the management of fungal infection. The prevalence of these species differed significantly in various geographic areas [4], [5], [6], [7], [8], [9], [10]. *Candida glabrata* was the most frequently isolated species in Western countries [4], [11], [12], whereas *C. tropicalis* predominated in Asia [5], [13], [14], [15]. For the treatment, azoles, echinocandins, polyenes, and 5-flucytosine are the four major classes of antifungal drugs. Due to low cost and less side effects, fluconazole has become one of the most commonly prescribed drugs.

Among the phenomena associated with azole resistance, ‘trailing’ describes the reduced but persistent growth that some isolates exhibit at drug concentrations above the minimum inhibitory concentrations (MICs) in broth dilution tests [16]. When the MIC of an isolate measured after 48 hours (h) incubation is approximately 4-fold higher than that at the 24 h point [17], the isolate is defined to have trailing growth. Thus, in the present study, isolates with fluconazole MICs≥64 mg/L or voriconazole MICs≥4 mg/L after 48 h incubation were considered to have reduced susceptibility to azole drugs.

In order to monitor the trends of species distribution and drug susceptibilities of yeast pathogens, the Taiwan Surveillance of Antimicrobial Resistance of Yeasts (TSARY) program was initiated in 1999 [18]. Subsequently, two more rounds of TSARY were conducted in 2002 and 2006. Previously, we found 23 of the 162 *C. tropicalis* isolates collected from TSARY in 1999 with fluconazole MICs≥64 mg/L to be closely related despite being collected from different hospitals throughout Taiwan [19], [20]. Furthermore, 5 of the 23 isolates exhibiting reduced susceptibility to fluconazole were from hospital N4 and all belonged to the same diploid sequence type (DST), DST140 (allele combination, 1, 3, 3, 17, 54, and 3 for *ICL1*, *MDR1*, *SAPT2*, *SAPT4*, *XYR1*, and *ZWF1a*, respectively). However, we did not find any evidence to suggest that these five isolates were transmitted horizontally from person to person [21]. In addition, 2 genetically closely related DST *C. tropicalis* strains, DST140 and DST98 (allele combination, 1, 3, 3, 17, 9, and 3), exhibited reduced susceptibility to fluconazole were identified in TSARY 1999 and 2006. Among them, 18 DST140 isolates were recovered from 10 different hospitals located in all 4 geographic regions in Taiwan and 7 DST98 ones were recovered from 4 different hospitals, two each in northern and southern Taiwan. There were also 3 DST149 (allele combination, 1, 44, 3, 7, 58, and 3) isolates exhibiting reduced susceptibility to fluconazole and from 3 hospitals located in northern Taiwan were identified. These results indicated that those DST strains exhibiting reduced susceptibility to fluconazole circulated widely in Taiwan from 1999 to 2006 and their presence was not a result of outbreaks in certain hospitals or geographic regions [21].

Exposure to azole compounds paves the way for the selection and enrichment of fungal isolates exhibiting reduced susceptibility to drugs, which may occur in patients receiving azole treatments. However, emergence of person to person transmission of isolates exhibiting reduced susceptibility to fluconazole during medical treatment is unlikely. Alternatively, the use of azole compounds in the environment may select organisms exhibiting reduced susceptibility to drugs, which may find their ways to human. *Candida tropicalis* is prevalent in organically enriched soil and aquatic environments [22] as well as in wild birds [23]. Thus, in the present study, we investigated the presence in soil of these *C. tropicalis* strains exhibiting reduced susceptibility to fluconazole, especially DST98, DST140, and DST149.

## Results

### *Candida tropicalis* isolates

A total of 56 *C. tropicalis* isolates (Table 1) analyzed in the present study were recovered from 26 of the 477 soil samples collected from 2006 to 2008 around Taiwan. The positive culture rates of *C. tropicalis* from various types of soil differed significantly. It was 85.7% (6/7) for petroleum-contaminated soil samplings, 44.8% (13/29) for agricultural fields, 38.5% (5/13) for sludge soil, and 0.5% (2/427) for forest soil. To identify whether multiple strains were present in a sample, three individual colonies (if present) of each representative morphotype in every soil sample were picked for subsequent workup. In the present study, 10 samples had only one *C. tropicalis* isolate recovered, 8 had 2, 5 had 3, 2 had 4, and 1 had 7(Table 1). All isolates were susceptible to amphotericin B. Among the 18 isolates with fluconazole MICs≥64 mg/L after 48 h incubation, 17 had voriconazole MICs≥4 mg/L as well. Nevertheless, they all exhibited trailing growth phenotype.

**Table 1:** Characteristics of *Candida tropicalis* isolates from soils (*N* = 56).

			minimum inhibitory concentration (mg/L)			
Isolate	Sample	DST	Amp 48 h	Flu 24 h	Flu 48 h	Vor 48 h
NHUE1	16	ND	0.5	0.25	0.5	0.0313
NHUE2	16	ND	0.5	0.25	0.5	0.0313
NHUE3	17	ND	0.5	0.25	0.5	0.0313
NHUE4	17	ND	0.5	0.25	0.5	0.0313
NHUE5	17	ND	0.25	0.25	0.5	0.0313
NHUE6	18	ND	0.5	0.25	0.5	0.0625
NHUE7	18	ND	0.5	0.25	0.5	0.0313
NHUE8	18	ND	0.5	0.25	0.5	0.0625
NHUE9	19	ND	1	0.25	0.5	0.0625
NHUE10	20	140	0.5	0.25	>64	>8
NHUE11	20	231	0.5	0.25	0.5	0.0313
NHUE12	21	ND	0.5	0.25	0.5	0.0313
NHUE13	21	ND	0.5	0.25	0.5	0.0313
NHUE14	21	ND	0.5	0.25	0.5	0.0313
NHUE15	26	ND	0.5	0.25	0.5	0.0313
NHUE16	27	ND	1	0.25	0.5	0.0625
NHUE17	28	149	0.5	0.5	>64	>8
NHUE18	28	149	0.5	0.5	>64	>8
NHUE19	28	149	0.5	0.5	>64	>8
NHUE20	7	ND	0.5	0.25	0.5	0.0625
NHUE21	4	226	0.5	0.25	64	0.0313
NHUE22	4	226	0.5	0.25	8	8
NHUE23	5	168	0.5	0.5	>64	>8
NHUE24	5	ND	0.5	0.25	0.5	0.0156
NHUE25	5	ND	1	2	4	0.0625
NHUE26	5	ND	0.5	2	2	0.0625
NHUE27	23	229	0.5	0.5	1	2
NHUE28	24	149	0.5	0.5	>64	>8
NHUE29	24	149	0.5	4	>64	>8
NHUE30	24	149	0.5	0.5	>64	>8
NHUE31	24	ND	0.5	0.25	0.5	0.0313
NHUE32	22	ND	0.5	0.125	1	0.25
NHUE33	22	149	0.5	2	>64	>8
NHUE34	22	149	0.5	0.125	>64	>8
NHUE35	25	ND	0.0313	0.125	0.125	ND
NHUE36	25	230	0.5	0.25	0.25	0.0156
NHUE37	6	183	0.5	0.5	>64	4
NHUE38	6	ND	0.0313	0.25	0.25	1
NHUE39	6	187	0.5	0.5	>64	>8
NHUE40	6	149	0.5	0.5	>64	>8
NHUE41	6	227	1	0.5	>64	>8
NHUE42	6	139	0.5	0.125	0.5	0.125
NHUE43	6	227	0.5	1	>64	>8
NHUE44	8	ND	0.5	0.25	0.5	0.0625
NHUE45	8	ND	0.5	0.25	0.5	0.0625
NHUE46	9	ND	0.5	0.25	0.5	0.0313
NHUE47	11	ND	0.5	0.25	0.5	0.0313
NHUE48	13	140	0.5	0.5	>64	>8
NHUE49	13	ND	1	0.125	0.25	0.0156
NHUE50	14	227	0.5	0.5	1	>8
NHUE51	15	ND	1	0.25	0.5	0.0625
NHUE52	15	191	0.25	0.25	1	0.5
NHUE53	12	ND	0.5	0.25	1	0.125
NHUE54	12	ND	0.5	0.25	0.5	0.0313
NHUE55	10	ND	0.5	0.25	0.5	0.0313
NHUE56	3	140	0.5	0.25	>64	4
ATCC 6258		ND	1	8	32	0.25
ATCC 90028		ND	0.5	1	2	0.0313
ATCC 22019		ND	0.5	0.125	0.125	0.0156
ATCC13803		ND	ND	0.5	0.5	ND

DST, diploid sequence type; h, hours; ND, not determined; amp, amphotericin B; flu, fluconazole; vor, voriconazole;

### Molecular typing

**Figure 1:**
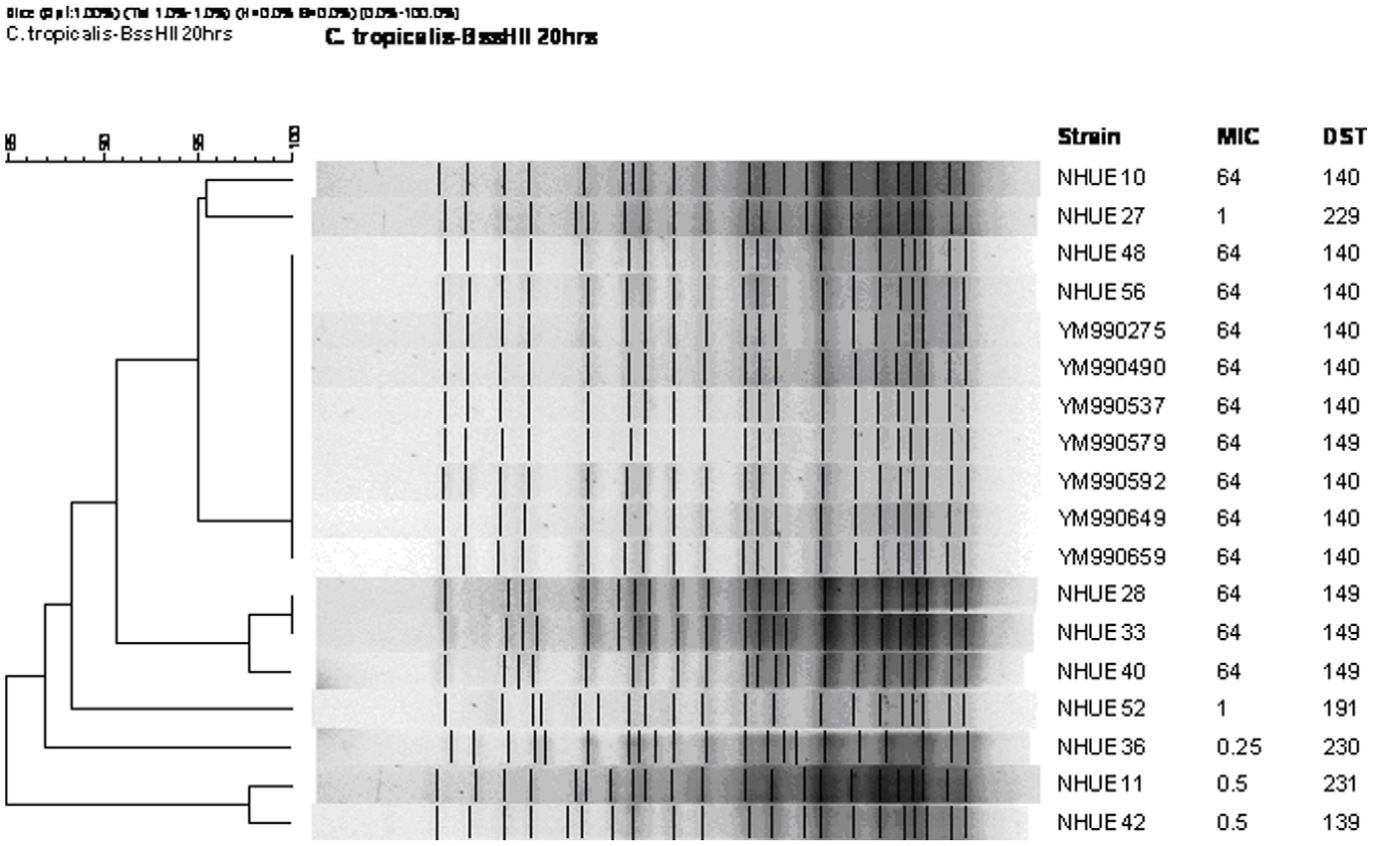
Dendrogram of 18 *Candida tropicalis* isolates from human and soil. The Dice coefficient was used to analyze the similarities between the PFGE band patterns. UPGMA was used for the cluster analysis. The position tolerance and optimization were set at 1%. A total of 7 isolates were recovered from human (YMs) and 11 from soil (NHUEs). MIC refers to fluconazole MIC (in mg/L). An MIC of 64 indicates isolates with reduced susceptibility.

To determine whether the *C. tropicalis* isolates with reduced susceptibility to fluconazole recovered from soil samples were genetically related to those from human, we applied pulsed field gel electrophoresis (PFGE) method to analyze their genetic relatedness. The 18 isolates that were investigated included 11 soil and 7 human isolates. The soil isolates comprised 6 isolates exhibiting reduced susceptibility to fluconazole (NHUE10, 28, 33, 40, 48, and 56) and 5 susceptible ones (NHUE11, 27, 36, 42, and 52). The 7 clinical isolates (YM990275, 490, 537, 579, 592, 649, and 659) all exhibited reduced susceptibility to fluconazole. As expected, all 6 DST140 isolates from human shared an indistinguishable PFGE pattern (Fig. 1). In addition, 2 isolates exhibiting reduced susceptibility to fluconazole from soil, NHUE48 and NHUE56, shared the same PFGE pattern with those human isolates. Among the remaining 4 soil isolates exhibiting reduced susceptibility to fluconazole, 3 (NHUE28, NHUE33, NHUE40), from different samples, shared at least 97% similarity. Furthermore, fluconazole susceptible isolates recovered from soil were not closely related to the ones exhibiting reduced susceptibility to fluconazole from the same areas, i.e. NHUE 11 vs. NHUE 10, NHUE 36 vs. NHUE 33, and NHUE 42 vs. NHUE 40. The fact that YM990579, a DST149 human isolate, also shared the indistinguishable PFGE pattern with the six DST140 human isolates, suggests that MLST has a higher discriminatory power than PFGE among *C. tropicalis* isolates. Therefore we further employed MLST to compare the genetic relatedness among all 18 soil isolates exhibiting reduced susceptibility to fluconazole. Among them, 9 from 4 soil samples (#6, 22, 24, and 28) were DST149 and 3 from 2 soil samples (#3 and 20) were DST140 (Table 1). In addition to DST140 and DST149, 10 additional DST patterns were identified from 13 isolates in the present study. They were DST139 (allele combination, 1, 4, 22, 23, 36, and 9), DST168 (allele combination, 1, 3, 3, 17, 57, and 3), DST183 (allele combination, 1, 20, 12, 17, 68, and 3), DST187 (allele combination, 1, 63, 2, 30, 69, and 25), DST191 (allele combination, 9, 54, 2, 10, 70, and 9), DST226 (allele combination, 1, 80, 27, 10, 44, and 9), DST227 (allele combination, 5, 54, 1, 47, 48, and 22), DST229 (allele combination, 1, 82, 3, 17, 57, and 3), DST230 (allele combination, 1, 4, 12, 10, 3, and 9), and DST231 (allele combination, 27, 9, 1, 43, 23, and 7). DST140 and DT168 are closely related. They are single locus variants and differ by only one SNP (nt 242) in the *XYR1* gene. Among the 6 tested fluconazole susceptible isolates, NHUE22 (MIC = 8 mg/L) was less susceptible than the other 5 (MIC ≦ 1 mg/L) and they all had their own unique DST. Interestingly, NHUE22 and NHUE21 were both from sample 4 and belonged to DST226.

### Growth curves

**Figure 2:**
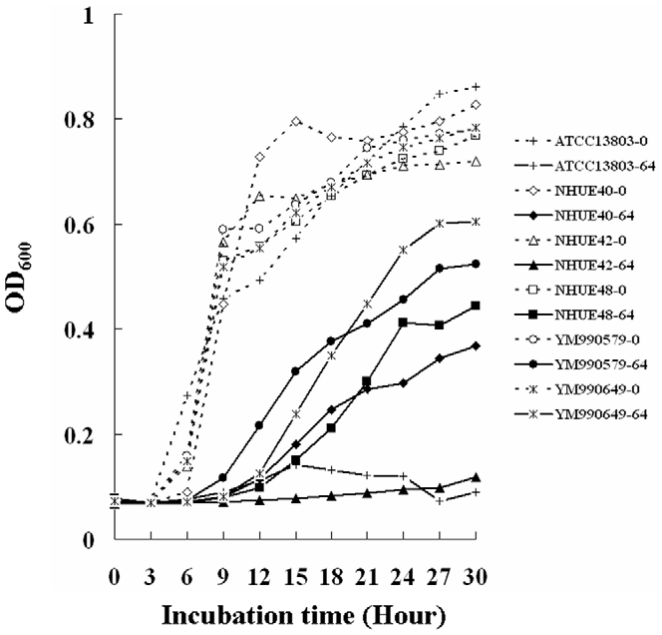
Growth curves of different *C. tropicalis* isolates with different fluconazole susceptibilities. Six isolates, ATCC13803 (rectangles), NHUE40 (diamonds), NHUE42 (triangles), NHUE48 (circles), YM990579 (crosses), and YM990649 (stars) were grown in the RPMI medium 1640 (Gibco BRL31800-022) in the absence (dot lines) or in the presence of 64 mg/L (solid lines) fluconazole.

All 18 soil isolates with fluconazole MICs≥64 mg/L after 48 h incubation, such as NHUE40, NHUE48, YM9900579, and YM990649, were able to grow in the presence of 64 mg/L fluconazole (Fig. 2). In contrast, the growth of the susceptible ones was inhibited, such as NHUE42 (Fig. 2, triangles solid line) and ATCC13803 (Fig. 2, crosses solid line). Hence, those isolates with fluconazole MICs≥64 mg/L definitely had reduced susceptibility to fluconazole than those isolates with lower fluconazole MICs.

### Susceptibilities to drugs used in agriculture

Since 17 of the 18 soil isolates exhibiting reduced susceptibility to fluconazole also had high voriconazole MICs, we selected 6 isolates to investigate whether isolates with reduced susceptibility to fluconazole from human and soil also had reduced susceptibility to other azole drugs used in agriculture. The relative growth of 6 isolates after incubation for 48 h is showed in Table 2. In the presence of 64 mg/L fluconazole, isolates exhibiting fluconazole reduced susceptibility, NHEU40, NHUE48, YM990579, and YM990649 grew better (66–100% of growth) than the fluconazole susceptible ones, ATCC13803 and NHUE42 (27–32% of growth), confirming our previous results obtained from broth microdilution method. All tested isolates grew poorly (10–11% of growth) in the presence of 64 mg/L penconazole and tebuconazole. Interestingly, the 4 isolates exhibiting fluconazole reduced susceptibility grew better than fluconazole susceptible ones in the presence of 4 mg/L penconazole (36–60% vs. 23–24% of growth), 4 mg/L tebuconazole (54–83% vs. 12–15% growth), 16 mg/L fluquinconazole (71–87% vs. 41–59% of growth), or 64 mg/L tridimenol (24–35% vs. 15–17% growth). Hence, isolates exhibiting fluconazole reduced susceptibility were also less susceptible than the fluconazole susceptible ones to other azole drugs used in agriculture.

**Table 2:** Relative growth of six *C. tropicalis* strains in the presence of different drugs.

Drug	none	Fluconazole		Penconazole		Tebuconazole		Tridimenol		Fluquinconazole	
Concentration (mg/L)		4	64	4	64	4	64	4	64	4	16*
ATCC13803	100#	**34** §	**27**	**23**	**11**	**15**	**10**	**49**	**15**	55	**41**
NHUE40	100	71	71	**36**	**10**	58	**10**	71	**34**	68	77
NHUE42	100	**30**	**32**	**24**	**11**	**12**	**10**	60	**17**	75	59
NHUE48	100	87	100	54	**11**	83	**11**	78	**35**	94	87
Ym990579	100	71	66	**40**	**10**	59	**10**	75	**35**	83	72
Ym990649	100	92	81	60	**11**	54	**11**	65	**24**	100	87

* Due to the solubility of fluquinconazole, the highest concentration of this drug in the present study was 16 mg/L instead of 64 mg/L.

# The growth of each isolate in the absence of drug was defined as 100% and the relative growth of each isolate in the presence of drugs was normalized accordingly.

§ Drugs capable of reducing the growth of cells by more than 50% are in bold, considered as susceptible to the concentrations of drugs.

## Discussion

*Candida tropicalis* is one of the most frequently isolated non-albicans *Candida* species from patients [8], [10], [24], [25]. Furthermore, *C. tropicalis* develops drug resistance in the presence of fluconazole much more rapidly than other *Candida* species [26], [27]. Our findings in the present study suggest that soil may be a potential source of *C. tropicalis* with reduced susceptibility to azole type of antifungal drugs.

The observation that 3 *C. tropicalis* isolates (NHUE28-30) with reduced susceptibility to fluconazole recovered from the same soil sample, #24, belonged to the same DST strain (DST149) suggests that they may be the progenies of the same strain. Nevertheless, one isolate (NHUE31) recovered from the same #24 soil sample was susceptible to fluconazole. On the other hand, of the 7 isolates (NHUE37-43) recovered from another soil sample, #6, 5 exhibited reduced susceptibility to fluconazole and belonged to 4 different DSTs (DST183, 187, 149, and 227). These results indicated that multiple strains of *C. tropicalis* exist in a single soil sample.

Long duration of drug exposure and high numbers of reproducing microorganisms contribute to the selection and/or enrichment of drug-resistant individuals. In fungi, drug resistance is more likely to be the outcome of sequential accumulation of adaptive mutations in the chromosomes [28], [29]. Azole-resistant isolates have been shown to emerge following microbial exposure to the drugs in patients and in agricultural settings. The observation that azole-resistant *Aspergillus fumigatus* isolates recovered from soil and compost were genetically related to clinical resistant isolates suggests the existence of an environmental route for developing drug resistance in fungi [30], [31], [32]. Similar to some antibiotic resistance in bacteria [33], [34], [35], fluconazole resistance in fungi also involves step-wise mutations affecting more than one genes [36], [37], [38]. Therefore, the *C. tropicalis* isolates from soil exhibiting trailing growth phenomena in the present study are prone to be resistant.

In the present study, we have found that *C. tropicalis* isolates with fluconazole MICs≥64 mg/L recovered from human and from soil, albeit from different geographic regions of Taiwan, shared indistinguishable PFGE patterns and belonged to the same DSTs. Whether the original development of *C. tropicalis* exhibiting reduced susceptibility to fluconazole occurred in human hosts or in the environments needs further investigation. However, our observation is consistent with the idea that medically important drug resistant isolates, not only bacteria but also fungi, exist in the environments.

Previous study has shown that in human immunodeficiency virus infected patients, *C. albicans* and other yeast species were cross-resistant to medical and agricultural azole drugs [39]. However, no *C. tropicalis* was recovered in that study. The observation that isolates with reduced fluconazole susceptibility were also more resistant than the susceptible ones to fluquinconazole and tridimenol but not to penconazole and tebuconazole (Table 2) may explain why the later two but not the former two are still used in agriculture in Taiwan. According to the data of “Domestic Manufacturers Production & Sale of Pesticides “ published by the Taiwan Crop Protection Industry Association, there is an increased amount of azole-type compounds used in the agriculture in Taiwan, from approximately 100 tons in 2005 to 145 tons in 2009 (about 45% increase). Since both efficient reproduction and spreading of resistant fungi in the environment are to be anticipated, we are confronted with the major challenge of drug-resistance development in environmental setting on a global scale. Hence, to reduce and prevent patients from acquiring azole-resistant *C. tropicalis* and other fungal species, it is advisable to take precaution against unnecessary use of azoles in not only clinical but also agricultural settings. The mechanisms contributing to the reduced susceptibility to azole drugs are under investigation. Further study should be performed to compare *C. tropicalis* isolates recovered from soils in the fields as well as those from farmers for genetic relatedness and susceptibility to various antifungal drugs.

## Materials and Methods

### Strain isolation

To isolate yeasts from the soil, we transferred approximately one gram of soil from each sample into a tube containing nine ml of sterile water and vortex-mixed. Diluted 0.2 ml of suspensions were plated onto acidified YMA (1% glucose, 0.5% peptone, 0.3% yeast extract, 0.3% malt extract, 1.5% agar, pH3.5) or Dichloran Rose Bengal Chloramphenicol agar (Merck, Darmstadt, Germany). After incubation at 24°C for 3 days, three (if present) representative colonies of each morphotype were picked for subsequent workup. All yeast isolates were identified by the sequences of the D1/D2 domain of the LSU rRNA genes as described by Lee et al. [40].

### Drug susceptibility testing

The MICs of amphotericin B (0.0313–16 mg/L), fluconazole (0.125–64 mg/L), and voriconazole (0.0156–8 mg/L) were determined by the same *in vitro* antifungal susceptibility testing established in our laboratory [41], [42] according to the guidelines of M27-A3 recommended by the Clinical and Laboratory Standards Institute [43]. RPMI medium 1640 (31800-022, Gibco BRL) was used for the dilution and growth of the yeast culture. Strains from American Type Culture Collection (ATCC), including *C. albicans* (ATCC 90028), *C. krusei* (ATCC 6258), and *C. parapsilosis* (ATCC 22019), were used as the standard controls. Growth of each isolate was measured by the Biotrak II plate spectrophotometric reader (Amersham Biosciences, Biochrom Ltd., Cambridge England) after incubation at 35°C for 24 h and 48 h.

The MIC of amphotericin B was defined as the minimum inhibitory concentrations of the drug capable of inhibiting cell growth. For susceptibility to amphotericin B, isolates with MIC≧2 mg/L were considered to be resistant and those with MIC≦1 mg/L were susceptible. The MIC for fluconazole and voriconazole was defined as the lowest concentration capable of reducing the turbidity of cells by more than 50%. Isolates with fluconazole MICs≥64 mg/L were considered as to have reduced susceptibility and ≤8 mg/L susceptible. The isolates with MICs in the range of 16–32 mg/L were referred to as susceptible-dose dependent (SDD). Isolates with voriconazole MICs≥4 mg/L were considered as with reduced susceptibility and ≤1 mg/L susceptible. The isolates with MIC 2 mg/L were referred to as SDD.

### Pulsed field gel electrophoresis (PFGE)

A total of 18 isolates were examined by PFGE, including 11 soil and 7 human isolates. The soil isolates comprised 6 (NHUE10, 28, 33, 40, 48, and 56) exhibiting fluconazole reduced susceptibility and 5 best matched susceptible ones isolated either from the same sample or from the same type of soil in the same area, NHUE11, 27, 36, 42, and 52 (vs. NHUE10, 28, 33, 40, and 48, respectively). The 7 human isolates, YM990275, 490, 537, 579, 592, 649, and 659, all exhibited reduced susceptibility to fluconazole. Pulsed field gel electrophoresis (PFGE) was performed based on our previous report [44] with some modifications. Briefly, organisms were grown on sabouraud dextrose agar (SDA) plate at 30°C overnight, after which the organisms were embedded in 1% agarose gel (SeaKem Gold agarose, FMC BioProducts). The plugs were subjected to cell lysis by lyticase at 37°C for 2 h, and then treated with proteinase K in 50 mM EDTA buffer at 50°C for 16 h. The genomic DNA was digested with 4 units of *Bss*HII at 37°C for 16 h. Electrophoresis was then performed with a CHEF MAPPER system (Bio-Rad, Hercules, Calif., USA) in 0.5×TBE buffer at 6 V/cm at 14°C with alternating pulses at an angle of 120 degrees in a 5–50 s pulse-time gradient for 20 h. A ladder of *Salmonella choleraesuis* subsp. *choleraesuis* (Smith) Weldin serotype Braenderup (ATCC BAA664) was used as the molecular weight marker. The PFGE pattern was analyzed by BioNumerics software (Version 4.5, Applied Maths, Kortrijk, Belgium). The position tolerance was set at 1% and optimization was set at 1%. The Dice coefficient was used to analyze the similarities of the band patterns. The unweighted pair group method using arithmetic averages (UPGMA) was used for cluster analysis.

### Multilocus sequence typing

A total of 18 isolates exhibiting reduced susceptibility to fluconazole and 5 best matched susceptible ones (either from the same sample or from the same type of soil in the same area), NHUE11, 27, 36, 42, and 52 (vs. NHUE10, 28, 33, 40, and 48, respectively) as well as NHUE 22 with MIC of fluconazole 8 mg/L, were analyzed. Based on our previous report [19], the DNA fragments of six genes: *ICL1*, *MDR1*, *SAPT2*, *SAPT4*, *XYR1*, and *ZWF1a* were sequenced for the analyses. The resulted sequences were aligned with BioNumerics 3.0 (Applied Maths, Kortrijk, Belgium) and compared with those in the database of *C. tropicalis* (http://pubmlst.org/website) to obtain the identifiers (DST).

### Growth curves

The Bioscreen C analyzer (Oy Growth Curves AB Ltd, Helsinki, Finland) was used to re-assess the growth of different isolates. Approximately 1×10^4^ cells in 200 µl of RPMI medium 1640 (Gibco BRL31800-022) were grown in the absence or in the presence of different drugs, including fluconazole, fluquinconazole, penconazole, tebuconazole, and tridimenol, at 35°C. According to the record from the Taiwan Corp Protection Industry Association, penconazole and tebuconazole but not fluquinconazole and tridimenol were currently in use for agriculture in Taiwan. The growths of cells were determined every 15 minutes. Due to the solubility of fluquinconazole, the highest concentration of this drug in the present study was 16 mg/L instead of 64 mg/L. The experiments were repeated twice with similar results.
